# Associations between health-related quality of life and physical function in older adults with or at risk of mobility disability after discharge from the hospital

**DOI:** 10.1007/s41999-021-00525-0

**Published:** 2021-06-09

**Authors:** Sylvia Sunde, Karin Hesseberg, Dawn A. Skelton, Anette Hylen Ranhoff, Are Hugo Pripp, Marit Aarønæs, Therese Brovold

**Affiliations:** 1 Institute of Physiotherapy, OsloMet—Oslo Metropolitan University (OsloMet), St. Olavs Plass, PO Box 4, 0130 Oslo, Norway; 2 Division of Medical Services, Diakonhjemmet Hospital, Vinderen, PO Box 23, 0319 Oslo, Norway; 3 School of Health and Life Sciences, Glasgow Caledonian University, Glasgow, UK; 4 Department of Clinical Science, University of Bergen, Bergen, Norway; 5 Faculty of Health Sciences, OsloMet—Oslo Metropolitan University (OsloMet), Oslo, Norway

**Keywords:** Activities of daily living, Aged, Hospitalisation, Quality of life

## Abstract

**Purpose:**

To optimise the treatment for older adults after hospitalisation, thorough health status information is needed. Therefore, we aimed to investigate the associations between health-related quality of life (HRQOL) and physical function in older adults with or at risk of mobility disability after hospital discharge.

**Methods:**

This cross-sectional study recruited 89 home-dwelling older people while inpatients within medical wards at a general hospital in Oslo, Norway. HRQOL [the Medical Outcome Study 36-Item Short-Form Health Survey (SF-36)] and physical function [the Short Physical Performance Battery (SPPB)] were measured a median of 49 [interquartile range (IQR) 26–116] days after discharge. Simple linear regression analyses were conducted, and multivariable regression models were fitted.

**Results:**

The mean age of the patients was 78.3 years; 43 (48.9 %) were females. Multivariable regressions showed positive associations between SPPB and the physical subscales {physical functioning [*B* (95% CI) 4.51 (2.35–6.68)], role physical [*B* (95% CI) 5.21 (2.75–7.67)], bodily pain [*B* (95% CI) 3.40 (0.73–6.10)] and general health [*B* (95% CI) 3.12 (1.13–5.12)]}. Univariable regressions showed no significant associations between SPPB and the mental subscales {vitality [*B* (95% CI) 1.54 (− 0.10–3.18)], social functioning [*B* (95% CI) 2.34 (− 0.28–4.96)], role emotional [*B* (95% CI) 1.28 (− 0.96–3.52)] and mental health [*B* (95% CI) 1.00 (− 0.37–2.36)]}.

**Conclusion:**

The results reinforce that physical function and physical HRQOL are strongly linked, and interventions improving physical function might improve physical HRQOL. However, this hypothesis would have to be tested in a randomised controlled trial.

**Trial registration:**

ClinicalTrials.gov. Registered 19 September 2016 (NCT02905383).

**Supplementary Information:**

The online version contains supplementary material available at 10.1007/s41999-021-00525-0.

## Key summary points

**Aim:**

To investigate if health-related quality of life (HRQOL) is associated with physical function in older adults with or at risk of mobility disability after hospital discharge.

**Findings:**

Better physical function (SPPB) was significantly associated with a higher level of physical HRQOL (SF-36 subscales physical functioning, role physical, bodily pain and general health).

**Message:**

The positive associations between physical function and physical HRQOL might indicate that the exercise interventions aiming to improve physical function might also improve physical HRQOL in this group of older adults.

**Supplementary Information:**

The online version contains supplementary material available at 10.1007/s41999-021-00525-0.

## Introduction

Physical function is a broad, multidimensional concept that was defined by Garber et al. [1] as ‘*the capacity of an individual to carry out the physical activities of daily living. Physical function reflects motor function and control, physical fitness, and habitual physical activity and is an independent predictor of functional independence, disability, morbidity, and mortality*’. Mobility is also considered a component of physical function and is the ability to move independently from one place to another [2]. Older adults who have been hospitalised often experience a decline in physical function in the postdischarge period [3, 4]. People with lower physical functions are especially at risk of decline [5, 6]. Guralnik et al. [7] tested the physical function of more than 4500 community-dwelling older adults using a follow-up assessment of mobility-related disability one–six years later; in the study, the authors defined mobility-related disability as the inability to walk 0.5 miles (805 m) or climb stairs without help, and physical function was measured using the Short Physical Performance Battery (SPPB) [8]. The authors found that compared with those showing the best performance (score of 10–12), the relative risks of mobility-related disability for those with scores of 4–6 ranged from 2.9 to 4.9, and the relative risk of mobility-related disability for those with scores of 7–9 ranged from 1.5 to 2.1 [7]. In the present study, an SPPB score ≤ 9 was used as the cut-off to identify older adults with or at risk of mobility disability.

Studies have reported that hospitalisation might negatively affect health-related quality of life (HRQOL) [9–11]. HRQOL can be defined as ‘*a multidomain concept that represents the patient’s overall perception of the impact of an illness and its treatment. An HRQL measure captures, at a minimum, physical, psychological (including emotional and cognitive), and social functioning*’ [12]. Furthermore, HRQOL is considered a key indicator of older people’s health status [13]. To facilitate empowerment and healthy ageing, it is important for older adults to preserve their self-care ability and manage their lives at home while having little need for health and social services [14].

In the general population of older adults and among older adults with different chronic diseases, previous research has established that HRQOL in older ages is associated with multiple variables, such as age, gender, living arrangement, comorbidities, body mass index (BMI), education and physical function [13, 15, 16]. Several studies have shown that poorer physical function is negatively associated with multiple domains of HRQOL [17–21]. A study by Brovold et al. [22] investigating the associations between HRQOL, physical fitness and physical activity in older adults recently discharged from the hospital found that the 6MWT was significantly associated with all domains of the SF-36. However, research on adults with or at risk of mobility disability after hospital discharge is scarce. The population of older adults admitted to the hospital is typically more frail, with more comorbidities and a less stable health status than the general older population [7, 23]. Hence, the associations between their HRQOL and physical function might be different from those of the general population of older adults. Knowledge about this relationship is important for the planning of interventions and allocation of health resources for this vulnerable population [13, 15].

To the best of our knowledge, the relationship between HRQOL and physical function in older adults with or at risk of mobility disability after hospital discharge has not been thoroughly established. Because of the complexity of the health challenges in this group, detailed information on their HRQOL is pivotal for optimising their health care services. Hence, the aim of the current study is to explore the relationships between HRQOL and physical function in older adults with or at risk of mobility disability after discharge from hospital. We hypothesise that there is an association between reduced physical function and poorer HRQOL in this population.

## Methods

### Study design, setting and participants

The current study has a cross-sectional design, and the data were retrieved from baseline measurements of a randomised controlled trial (RCT) investigating the effects of a multicomponent high-intensity exercise intervention on physical function and HRQOL in older adults with or at risk of mobility disability after discharge from hospital [23]. The participants were recruited from medical units for stroke, geriatrics, cardiology, pulmonology and infectious diseases at a general hospital in Oslo, Norway. The recruitment period ran from September 2016 to May 2019.

To be eligible for inclusion, the participants had to live independently in the community before admission, be at risk of mobility disability (defined as a SPPB score ≤ 9 while inpatient) [8], ambulate independently (walking aid permitted) and understand the Norwegian language. Furthermore, they had to be assessed by a doctor as eligible for the intervention (in the RCT) based on the standards from the American Heart Association [24]. The participants were considered noneligible if they had any moderate or severe cognitive disorder (score on Mini Mental State Examination < 20), if they had life expectancy less than eight months or if they exercised regularly more than twice a week at a fitness centre or in a structured exercise programme prior to admission. Baseline testing was conducted by a physiotherapist at a hospital outpatient clinic after the participants had returned home and hospital-initiated rehabilitation was completed. Time since discharge was a median of (IQR) 49 (26–116) days.

The Regional Ethics Committee for Medical Research approved the study (REK 2015/2432), and the trial was registered at ClinicalTrials.gov in September 2016. The participants provided written informed consent, and the project was conducted according to the World Medical Association Declaration of Helsinki. The STROBE guidelines have been followed in our report on the design, analysis and presentation of data.

### Outcome measures

*Characteristics* such as age (years), sex, living alone or with someone, postsecondary education (bachelor’s degree or higher), number of comorbidities at the time of discharge, length of hospital stay and days from discharge to baseline testing were recorded from the participants’ hospital notes and by asking the participants.

*Health-related quality of life* was measured by the Medical Outcome Study 36-Item Short-Form Health Survey, version 2 (SF-36) [25]; this is a generic questionnaire that has been translated into Norwegian, and Norwegian reference values have been established [26]. The 36 items in the SF-36 are grouped into eight subscales separated into two main groups: physical and mental health. The physical health scales include physical functioning, role limitations because of physical problems, bodily pain and general health perception. The mental health scales include vitality, social functioning, role limitations because of emotional problems and mental health. Each subscale score is transformed into percentages from 0 (worst) to 100 (best) [25].

*Physical function* was assessed by measurements of balance, gait speed, functional capacity, muscle strength and BMI.

The SPPB is a performance-based test that evaluates balance, functional mobility (gait speed) and muscle strength by examining an individual’s ability to stand with feet together in side by side, semitandem and tandem positions, time to walk four metres and time to perform five repeated chair stands [8]. Based on performance, each of the three tests is scored between 0 and 4, leaving a maximum score of 12 for those individuals performing at the highest levels. Reference values for older people in Norway have been established [27].

The Berg Balance Scale (BBS) is a measure of static and dynamic balance that comprises 14 observable tasks frequently encountered in everyday life. The patient’s ability to perform the 14 tasks was scored on a 5-point scale from 0 (cannot perform) to 4 (normal performance). The sum score ranges from 0 (worst) to 56 (best) [28].

The functional capacity (endurance) was measured by the six-minute walk test (6MWT), a submaximal test in which the distance walked over six minutes is assessed [29]. The participants were instructed to walk as fast as possible for six minutes on a flat hard surface back and forth around two cones placed 30 m apart. To maintain physical independence, women and men in the 75–79-year-old age group should be able to walk at least 503 m (550 yards) and 530 m (580 yards), respectively [30].

Muscle strength (grip strength) was measured using a Jamar digital dynamometer [31]. Low grip strength is a powerful predictor of death, the development of disability, prolonged length of hospital stay and low HRQOL [32]. Grip strength < 27/ < 16 kg is suggested as a cut-off point for low strength when identifying sarcopenia in older men/women [32].

Weight and BMI were measured with a Tanita BC-418 body composition analyser for participants without a pacemaker (contraindication). A regular body scale was used for patients with a pacemaker, and BMI was calculated (kg/m^2^). Height was measured using a stadiometer, with the measurement taken to the nearest centimetre. BMI does not distinguish between fat mass and muscle mass and may be a poor indicator of fat percentage in older people [33]. Despite this lack of sensitivity, relatively consistent links between BMI and physical disability or functional limitations have been established [34].

### Statistical analyses

Statistical analyses were conducted using IBM SPSS Statistics 25.0 (SPSS Inc., Chicago, IL). *P* values < 0.05 were considered statistically significant, and all tests were two sided. The normality of the distributions was examined graphically by histograms and Q–Q plots and by comparing the mean with the median. Data are described as the means and standard deviations (SD) for normally distributed continuous variables and median and quartiles (25, 75) for a continuous variable that was skewed (length of stay). Categorical variables are described with proportions and percentages. The independent samples *t*-test and the *χ*^2^ test for independence were used to identify significant differences in age and gender among the participants who were included or excluded.

Simple regression analyses were used to explore the associations between each of the SF-36 subscales (dependent variables) and the background variables (age, sex, postsecondary education, living alone or with someone, number of comorbidities at the time of discharge, length of stay and number of days between discharge and baseline testing), BBS, 6MWT, grip strength and SPPB. Further, age, sex and variables with the strongest association (*P* < 0.05) with the dependent variable (SF-36 subscale) from the univariable analyses were assessed in multivariable linear regression models. Regression model assumptions were examined graphically and analytically. No missing values were replaced. Floor and ceiling effects were considered by examining whether more than 20% of the participants had the lowest or highest possible score.

## Results

### Participant characteristics

Five hundred and thirty-eight participants were screened for eligibility, of which 89 were included. The most common reasons for declining to participate were regular physical training at fitness centres, being too busy helping kin or others, not wanting regular appointments twice a week, travelling time/logistics and spending time abroad. There were no statistically significant differences between the participants who were included in the study and those who were excluded regarding gender and age. Recruitment was stopped before the estimated sample size was reached because of slow recruitment and a limited time frame.

Table 1 shows the characteristics of the total sample. The study sample comprised 43 women and 46 men. One man withdrew from the study and requested that we delete the data concerning him. Mean (SD) age was 78.3 (± 5.5) years. The most common hospital admission diagnoses (ICD-10) of the included patients were as follows: diseases of the circulatory system (*n* = 31, 35.2%), diseases of the respiratory system (*n* = 14, 15.9%), diseases of the genitourinary system (*n* = 9, 10.2%), diseases of the eye and adnexa/ear and mastoid process (*n* = 6, 6.8%), diseases of the nervous system (*n* = 5, 5.7%), diseases of the musculoskeletal system and connective tissue (*n* = 5, 5.7%) and mental and behavioural disorders (*n* = 4, 4.5%). Eighty-one patients (92%) had two or more comorbidities. The mean (SD) grip strength for the men was 32.5 (7.9) kg and 19.8 (4.5) kg for the women. Seven (16.3%) out of the women and 10 (22.2%) of the men had grip strength below 16 kg and 27 kg, respectively. Eight of the 43 (18.6%) female participants walked at least 503 m (550 yards) on the 6MWT. Eight out of the 45 (17.8%) male participants walked at least 530 m (580 yards) on the 6MWT. No floor and ceiling effects occurred, except for the SF-36 subscale social functioning, where 24% of the men and 26% of the women achieved the maximum score of 100 points.

**Table 1:** Characteristics of the study sample. ﻿Means, standard deviations (SD), numbers and percentages﻿

	Total (*N* = 88)
Characteristics	
Age in years, mean (SD)	78.3 (5.5)
Sex, female %	48.9
Living alone, *n* (%)	45 (51.1)
Postsecondary education, *n* (%)	53 (60.2)
Length of stay in days, median (IQR)	2 (1–4)
Comorbidities, mean (SD)	4.5 (2.3)
Physical function	
Short physical performance battery, mean (SD)	8.7 (2.3)
Grip strength, mean (SD)	26.3 (9.1)
Berg Balance Scale, mean (SD)	48.9 (6.8)
Six-min walk test *m*, mean (SD)	387.4 (115)
Body mass index, mean (SD)	26.9 (5.4)
Health-related quality of life (SF-36)	
Physical functioning, mean (SD)	58.9 (23.3), *n* = 88
Role physical, mean (SD)	47.0 (27.8), *n* = 83
Bodily pain, mean (SD)	56.6 (25.9), *n* = 83
General health, mean (SD)	52.2 (21.6), *n* = 85
Vitality, mean (SD)	44.9 (17.5), *n* = 86
Social functioning, mean (SD)	67.5 (28.1), *n* = 87
Role emotional, mean (SD)	59.4 (23.2), *n* = 83
Mental health, mean (SD)	67.7 (14.4), *n* = 85

*SF-36*  the Medical Outcome Study 36-Item Short-Form Health Survey, *CI* confidence interval, *SD* standard deviation, *IQR* interquartile range, *N* number, *Postsecondary education* bachelor’s degree or higher (yes/no)

### Regression analysis

The simple regression analysis showed statistically significant associations between the four physical subscales of the SF-36 (physical functioning, role physical, bodily pain and general health) and SPPB. There were no statistically significant associations between SPPB and the mental subscales of the SF-36 (vitality, social functioning, role emotional and mental health). Grip strength was significantly associated with the subscales of physical functioning and bodily pain. Comorbidity was significantly associated with the subscales of physical functioning and bodily pain, and age was significantly associated with the subscale of role physical. Finally, having a postsecondary education was significantly associated with higher scores on physical functioning, bodily pain and vitality. There were no statistically significant associations between any of the subscales of SF-36 and the variables of sex, living alone or with someone, BMI, length of stay, and number of days from discharge to baseline testing. Further results from the univariable regressions are presented in the supplementary material (Table 3).

Table 2 presents the results of the multivariable regressions of the eight subscales of the SF-36 on age and sex and any additional variable that was significantly associated with the respective subscale in the univariable analysis (SPPB, grip strength, education and/or comorbidity). There was a statistically significant positive association between the four physical subscales of the SF-36 (physical functioning, role physical, bodily pain and general health) and SPPB. Finally, having a postsecondary education remained significantly associated with higher scores on physical functioning and bodily pain. The BBS and the six-minute walk test were highly correlated (> 0.6) with the SPPB, so these variables were excluded from the final models. The multivariable models accounted for 0–32% of the variance.

**Table 2:** Multivariable regression of the SF-36 (HRQOL) on characteristics and physical function

SF-36 (HRQOL)	Characteristics	Standardised *β*	*P* value	*B* (95%CI)	Adjusted *R*^2^
Physical functioning	Age	0.05	0.619	0.20 (− 0.59–0.98)	0.319
	Sex	0.16	0.237	7.61 (− 5.09–20.31)	
	Education	0.24	0.012	11.51 (2.62–20.39)	
	Comorbidity	− 0.05	0.621	− 0.49 (− 2.46–1.48)	
	SPPB	0.44	< 0.001	4.51 (2.35–6.68)	
	Grip strength	0.17	0.254	0.44 (− 0.32–1.21)	
Role physical	Age	− 0.16	0.118	− 0.81 (− 1.84–0.21)	0.193
	Sex	0.07	0.485	3.92 (− 7.20–15.03)	
	SPPB	0.43	< 0.001	5.21 (2.75–7.67)	
Bodily pain	Age	0.15	0.153	0.70 (− 0.27–1.67)	0.213
	Sex	− 0.0	0.992	− 0.08 (− 15.8–15.6)	
	Education	0.23	0.033	11.95 (0.99–22.92)	
	Comorbidity	− 0.14	0.190	− 1.62 (− 4.05–0.82)	
	SPPB	0.30	0.013	3.40 (0.73–6.10)	
	Grip strength	0.06	0.738	0.16 (− 0.79–1.11)	
General health	Age	0.21	0.050	0.83 (0.00–1.66)	0.099
	Sex	0.07	0.536	2.81 (− 6.20–11.82)	
	SPPB	0.33	0.003	3.12 (1.13–5.12)	
Vitality	Age	− 0.04	0.720	− 0.13 (− 0.82–0.57)	0.025
	Sex	− 0.10	0.388	− 3.39 (− 11.15–4.38)	
	Education	0.20	0.075	7.18 (− 0.73–15.10)	
Social functioning	Age	0.04	0.694	0.22 (− 0.90–1.34)	− 0.01
	Sex	− 0.10	0.380	− 5.38 (− 17.51–6.75)	
Role emotional	Age	− 0.02	0.882	− 0.07 (− 1.02–0.88)	− 0.02
	Sex	− 0.10	0.388	− 4.48 (− 14.76–5.80)	
Mental health	Age	0.08	0.482	0.21 (− 0.37–0.79)	− 0.02
	Sex	− 0.03	0.825	− 0.70 (− 7.01–0.79)	

Model fit reported by *R*^2^-adjusted. *N* = 88, except SF-36 (*n* = 83–88)

*SPPB* Short Physical Performance Battery, *CI* confidence interval, *HRQOL* health-related quality of life, *B* unstandardised beta

## Discussion

To the best of our knowledge, this is the first study exploring the relationship between HRQOL and physical function in older adults with or at risk of mobility disability after discharge from hospital. A multivariable regression analysis showed significant positive associations between the physical subscales of the SF-36 (HRQOL) and SPPB. The results indicate that a higher score on SPPB is associated with a better score on physical HRQOL. No significant associations were found between SPPB and the mental subscales of the SF-36.

Our results are partly in line with a study by Brovold et al. [22], where the authors found a significant positive association between all the subscales of the SF-36 and physical function (the 6MWT) in a Norwegian sample of 115 independent older people recently discharged from the hospital. However, in contrast to this study, the present study found no significant associations between physical function (SPPB) and the mental subscales. This discrepancy could be because of the more fit sample in the study by Brovold et al. [22], or it may be a result of the rather low sample size in the present study. The lack of significant associations between SPPB and the mental subscales of the SF-36 corresponds well with the results of a systematic review and meta-analysis investigating the effect of physical activity on HRQOL in older community-dwelling adults, where a significant (small to moderate) standardised effect size in improvement was found solely for the SF-36 subscale physical functioning as a result of physical activity [35]. Contrary to this, a recent systematic review and meta-analysis investigating the effect of resistance training on HRQOL in older adults found that resistance training significantly increased the subscales of physical functioning, bodily pain, general health, vitality and mental health in the initial analysis, and after removing a single study significant improvements were found for all the eight subscales of SF-36 [36]. This might indicate that the associations between HRQOL and physical function and the effect of interventions to improve HRQOL and physical function depend on the exercise mode and the population being investigated. Further, because of the multidimensional nature of HRQOL, its associations with SPPB can be affected in multiple ways [12, 13, 15].

Furthermore, the results of the present study are partly in line with a study by Asmus-Szepesi et al. [37] from the Netherlands. The authors aimed to validate the predictive ability of a screening questionnaire to identify older patients at risk for functional dependence; they did not investigate the associations between HRQOL and physical function, but they found that older people at low risk for functional dependence at hospital admission reported significantly higher HRQOL on almost all subscales of the SF-12 (a shorter version of the SF-36), both at admission and three and 12 months after admission, compared with people at high risk [37]. However, they measured physical function with questionnaires, and comparisons should be done with caution because self-reported and performance-based measures of physical function have been shown to capture different aspects of the construct [38]. Similarly, a study by Parlevliet et al. [39] investigated the independent associations between HRQOL at admission and risk of functional decline three months after admission in a group of 473 acutely hospitalised older people [39]; they used the EuroQol-5D to measure HRQOL, finding that participants with higher scores on the HRQOL at admission had a lower risk for functional decline three months later.

As mentioned in the introduction, the literature on studies exploring the associations between HRQOL and physical function in the population of older people after hospitalisation is scarce. When comparing our results with studies investigating associations in different populations of older adults, the results correspond well with the results in a study by Davis et al. [21]; Davis et al. [21] applied the EQ-5D-3L to assess HRQOL and reported a statistically significant association between SPPB and HRQOL in community-dwelling older adults. Furthermore, the results in the present study are consistent with a recent study by Bjerk et al. [19], who found significant associations between physical function (BBS and gait speed) and one of the physical subscales of the SF-36 (physical functioning) in a sample of older fallers receiving home care. Further, Bjerk et al. [19] did not show any significant association between physical function and the other seven subscales of the SF-36, but the authors reported that the sample size could have been too low to detect other associations.

Our results correspond well with a recent study on associations between HRQOL (SF-36) and physical function in a sample of 149 home-dwelling Norwegian women with osteoporosis and vertebral fracture, where physical function (walking speed) was significantly associated with the physical subscales of physical functioning, role physical and general health [20]. However, contrary to our findings, they did not find a significant association between physical function and bodily pain, but they did find a significant association between physical function and the subscales of social functioning and role emotional. Further, the authors reported no significant association between the other measures of physical function (functional reach, 30 s sit to stand and arm curls) and the mental subscales of the SF-36 [20]. Regarding the physical subscales, they found a significant association between functional reach and physical functioning and role physical and between 30 s sit to stand and physical functioning.

In contrast to our hypothesis, grip strength was not associated with any of the SF-36 subscales after controlling for age, sex and any additional variables that were significantly associated with the dependent variable in the crude analysis. These results are in contrast with a study by Sayer et al. [40], who found significant associations between grip strength and the subscales of physical functioning and general health, here after controlling for age, height, weight adjusted for height, self-reported walking speed, social class, smoking, alcohol consumption and known comorbidity in community-dwelling men and women (aged 59–73 years). Further, Halaweh et al. [41] found significant correlations between grip strength and the functioning and subjective well-being domains of EQ-5D-5L in older adults aged 60 years or older (mean age 68.2). Finally, Wanderley et al. [42] reported that community-dwelling older adults (60–83 years, mean age 68.0) with superior grip strength were more likely to score higher on the subscales of role physical and vitality. The lack of consistency between our findings and the results found in the mentioned studies could be because of the higher mean age in our sample, in addition to the higher mean grip strength in the studies by Sayer et al. [40] and by Wanderley et al. [42] compared with the means for the men and women in the present study.

An interesting finding is that the number of comorbidities was significantly associated with the subscales of physical functioning and bodily pain in the univariable regression analysis, but these associations were no longer significant after controlling for age, sex, education, SPPB and grip strength in the multivariable regression analysis. Similar results were found by Brovold et al. [22], suggesting that interventions addressing mobility disabilities may provide important health benefits to this population [17]. Further, the multivariate regressions showed statistically significant positive associations between education and the subscales of physical functioning and bodily pain, with 11.5 points and 12.0 points higher scores, respectively, when comparing the participants with postsecondary education to the participants who did not have such high education. This is in line with a recent study from Sweden, which reported that high education and good personal economy were associated with better health compared with people with lower education and financial strains [43].

The rather low adjusted *R*^2^ in our sample suggests that HRQOL is associated with numerous additional factors that should be considered in future research [12]. A recent study [13] investigating a sample of functionally independent community-dwelling older people from Spain found that polypharmacy, the presence of sensory impairment, not being engaged in cognitively stimulating activities or in group social activities, a low level of social support and the presence of obstacles in the closest home environment were significantly associated with a poor HRQOL.

Previous research has shown that hospitalisation represents a high-risk event for older adults regarding a decline in HRQOL and physical function [3, 4, 9, 10, 37]. When comparing the study sample in the present study with age-matched Norwegian reference values [26], the participants in the study sample scored substantially worse on both SPPB and HRQOL (SF-36); these results reinforce that older patients with or at risk of mobility disability while hospitalised represent older people with an increased risk of reduced independence and HRQOL.

Studies have shown that rehabilitation and exercises in hospitals or immediately after discharge can improve HRQOL and physical function in older people [44–46], but the evidence is not robust. Further research should investigate the effects of interventions aiming to improve HRQOL and physical function in this population. Physiotherapists and other health care personnel should motivate patients to be as physically active as possible and exercise as much as tolerated while being an inpatient to prevent declines in physical function during hospitalisation. Further, patients with or at risk of mobility disability should receive postdischarge rehabilitation at home, as an outpatient or in an institution to address these issues.

The present study has several limitations. The participants in the current study were recruited to an eight-month randomised controlled exercise trial [23]. They were recruited during their inpatient stay; however, the time point of baseline assessments was not possible to standardise because of the heterogeneity of the participants. Some of the participants were baseline tested after they had completed hospital initial rehabilitation, while other participants needed more time to become medically fit to exercise. This difference in the time from discharge to baseline testing may have had an impact on the results. However, no significant associations were found between the number of days from discharge to baseline testing and the subscales of the SF-36. Furthermore, by allowing the participants to enrol in the study when they were ready, we were able to increase the sample size. In addition, this might have increased the external validity of the study, with the study sample being representative of the underlying population [47].

We experienced that it was difficult to recruit participants, and a large portion of the eligible patients declined to participate. According to Buurman [47], this problem is frequently encountered in studies recruiting acutely hospitalised older people. The inclusion rate might have implications for the generalisability of the study results. We found no significant differences in age or sex between those who declined participation and those who accepted inclusion. Unfortunately, patient information protection laws prohibited us from further analysis, and other variables (characteristics, physical function and HRQOL) could have differed. As the adjusted *R*^2^ indicates, HRQOL is a multidimensional concept [12, 13, 15], and variables other than the ones we have measured (e.g. depression, polypharmacy, the presence of sensory impairment, not being engaged in cognitively stimulating activities or in group social activities, a low level of social support) may explain the variability in the participants’ SF-36 subscale scores. Further studies should measure as many of these variables as possible, and the failure to include these variables can be considered a limitation with the present study. In addition, data on premorbid physical function and cognitive status should have been provided to better characterise the sample.

Furthermore, the participants who were included had agreed to participate in a multicomponent high-intensity exercise trial aiming to increase their HRQOL and physical function. This may have led to the self-selection of the most fit and engaged part of the population, and the associations observed in the present study may not be applicable to the overall population of older people with or at risk of mobility disability while being inpatients. In addition, the participants were recruited from only one hospital, and the study sample may not be representative of the general population of older people in Norway. Finally, the present study is cross-sectional, which prevents causal relations from being established. Nevertheless, cross-sectional studies add information to the understanding of which factors have an effect on health and are important for generating hypotheses for future research [48]. One strength of the present study is that it contributes new knowledge on HRQOL and physical function, as well as the relationship between these variables, in this population of older people with or at risk of mobility disability, a group of people who are often excluded from studies despite being at high risk for multiple negative outcomes.

In conclusion, the present study demonstrates that low physical function after discharge from the hospital was significantly associated with reduced physical HRQOL. Further, these older patients scored substantially worse on HRQOL and SPPB compared with reference values for the general population of older people in Norway. Therefore, older people with or at risk of mobility disabilities while inpatients require extra attention to mitigate poor health outcomes. Further research should investigate the effects of interventions aiming to improve HRQOL and physical function in this population.

## Supplementary Information

Below is the link to the electronic supplementary material.

Supplementary file1 (DOCX 67 kb)

## Data Availability

The datasets used and/or analysed during the current study are available from the corresponding author on reasonable request.
