# Supplementary material for: Associations between health-related quality of life and physical function in older adults with or at risk of mobility disability after discharge from the hospital
Source: Eur Geriatr Med. 2021 Jun 9;12(6):1247–56. doi: 10.1007/s41999-021-00525-0 (PMC8626396; doi:10.1007/s41999-021-00525-0)
Supplement: Supplementary file 1 — Supplementary file1 (DOCX 67 kb) [file 41999_2021_525_MOESM1_ESM.docx]

**Supplementary material**

**Table 3** Univariable regression of the SF-36 (HRQOL) on characteristics and physical function

| SF-36 (HRQOL): | Characteristics | Standardised β | *P*-value | B (95%CI) |
| --- | --- | --- | --- | --- |
| Physical Functioning | Age | -0.00 | 0.986 | -0.01 (-0.92–0.91) |
|  | Sex | -0.08 | 0.440 | -3.87 (-13.76–6.03) |
|  | Education | 0.31 | 0.003 | 14.60 (4.95–24.24) |
|  | Comorbidity | -0.28 | 0.007 | -2.86 (-4.93–-0.79) |
|  | Living condition | 0.01 | 0.936 | 0.42 (-9.52–10.33) |
|  | Length of stay | 0.011 | 0.297 | 0.240 (-0.22–0.69) |
|  | Days since discharge | -0.10 | 0.335 | -0.04 (-0.13–0.04) |
|  | BMI | -0.07 | 0.528 | -0.29 (-1.21–0.62) |
|  | SPPB | 0.54 | < 0.001 | 5.53 (3.67–7.38) |
|  | Grip strength | 0.31 | 0.003 | 0.81 (0.29–1.3) |
|  | Berg Balance Scale | 0.47 | < 0.001 | 1.59 (0.94–2.24) |
|  | 6-min walk test | 0.65 | < 0.001 | 0.13 (0.10–0.16) |
| Role Physical | Age | -0.22 | 0.048 | -1.11 (-2.21–-0.01) |
|  | Sex | 0.04 | 0.699 | 2.38 (-9.84–14.61) |
|  | Education | 0.11 | 0.308 | 6.40 (-6.02–18.82) |
|  | Comorbidity | -0.13 | 0.236 | -1.59 (-4.23–1.06) |
|  | Living condition | -0.06 | 0.605 | -3.19 (-15.40–9.03) |
|  | Length of stay | 0.08 | 0.503 | 0.19 (-0.37–0.75) |
|  | Days since discharge | -0.09 | 0.442 | -0.04 (-0.15–0.06) |
|  | BMI | 0.16 | 0.149 | 0.82 (-0.30–1.94) |
|  | SPPB | 0.44 | < 0.001 | 5.34 (2.90–7.78) |
|  | Grip strength | 0.21 | 0.061 | 0.63 (-0.03–1.3) |
|  | Berg Balance Scale | 0.35 | 0.001 | 1.42 (0.57–2.27) |
|  | 6-min walk test | 0.43 | < 0.001 | 0.10 (0.06–0.15) |
| Bodily Pain | Age | 0.15 | 0.172 | 0.72 (-0.32–1.76) |
|  | Sex | -0.17 | 0.131 | -8.62 (-19.87–2.63) |
|  | Education | 0.33 | 0.003 | 17.15 (6.14–28.17) |
|  | Comorbidity | -0.32 | 0.003 | -3.61 (-5.96–-1.25) |
|  | Living condition | -0.20 | 0.075 | -10.14 (-21.33–1.04) |
|  | Length of stay | 0.11 | 0.324 | 0.26 (-0.26–0.78) |
|  | Days since discharge | -0.07 | 0.505 | -0.03 (-0.13–0.07) |
|  | BMI | -0.11 | 0.328 | -0.52 (-1.57–0.53) |
|  | SPPB | 0.38 | < 0.001 | 4.35 (2.02–6.69) |
|  | Grip strength | 0.27 | 0.014 | 0.77 (0.16–1.38) |
|  | Berg Balance Scale | 0.25 | 0.024 | 0.95 (0.13–1.77) |
|  | 6-min walk test | 0.38 | < 0.001 | 0.09 (0.04–0.13) |
| General Health | Age | 0.16 | 0.135 | 0.65 (-0.21–1.50) |
|  | Sex | 0.00 | 0.991 | 0.05 (-9.33–9.44) |
|  | Education | 0.04 | 0.709 | 1.80 (-7.78–11.38) |
|  | Comorbidity | -0.09 | 0.398 | -0.87 (-2.90–1.17) |
|  | Living condition | -0.09 | 0.407 | -13.26–5.43) |
|  | Length of stay | 0.02 | 0.857 | 0.04 (-0.39–0.47) |
|  | Days since discharge | -0.07 | 0.532 | -0.03 (-0.11–0.06) |
|  | BMI | 0.01 | 0.920 | 0.04 (-0.82–0.91) |
|  | SPPB | 0.30 | 0.006 | 2.82 (0.83–4.81) |
|  | Grip strength | 0.16 | 0.133 | 0.39 (-0.12–0.91) |
|  | Berg Balance Scale | 0.24 | 0.026 | 0.77 (0.10–1.44) |
|  | 6-min walk test | 0.28 | 0.010 | 0.05 (0.01–0.09) |
| Vitality | Age | -0.00 | 0.969 | -0.01 (-0.71–0.68) |
|  | Sex | -0.15 | 0.176 | -5.14 (-12.62–2.35) |
|  | Education | 0.22 | 0.038 | 7.98 (0.44–15.51) |
|  | Comorbidity | 0.04 | 0.752 | 0.26 (-1.39–1.91) |
|  | Living condition | -0.05 | 0.624 | -1.87 (-9.43–5.69) |
|  | Length of stay | 0.02 | 0.870 | 0.03 (-0.32–0.38) |
|  | Days since discharge | 0.10 | 0.875 | 0.03 (-0.04–0.09) |
|  | BMI | 0.02 | 0.885 | 0.05 (-0.65-–0.75) |
|  | SPPB | 0.20 | 0.066 | 1.54 (-0.10–3.18) |
|  | Grip strength | 0.10 | 0.339 | 0.20 (-0.22–0.62) |
|  | Berg Balance Scale | -0.03 | 0.822 | -0.06 (-0.63–0.50) |
|  | 6-min walk test | 0.11 | 0.337 | 0.02 (-0.02–0.05) |
| Social Functioning | Age | 0.06 | 0.609 | 0.29 (-0.82–1.39) |
|  | Sex | -0.10 | 0.347 | -5.69 (-17.66–6.28) |
|  | Education | 0.18 | 0.103 | 10.04 (-2.06–22.13) |
|  | Comorbidity | 0.01 | 0.932 | 0.11 (-2.51–2.73) |
|  | Living condition | 0.01 | 0.947 | 0.40 (-11.63–12.43) |
|  | Length of stay | -0.16 | 0.138 | -0.41 (-0.96–0.14) |
|  | Days since discharge | 0.01 | 0.921 | 0.01 (-0.10–0.11) |
|  | BMI | 0.08 | 0.490 | 0.39 (-0.27–1.50) |
|  | SPPB | 0.19 | 0.079 | 2.34 (-0.28–4.96) |
|  | Grip strength | 0.11 | 0.303 | 0.35 (-0.32–1.01) |
|  | Berg Balance Scale | -0.0 | 0.976 | -0.01 (-0.90–0.88) |
|  | 6-min walk test | 0.10 | 0.382 | 0.02 (-0.03–0.08) |
| Role Emotional | Age | -0.00 | 0.970 | -0.02 (-0.96–0.92) |
|  | Sex | -0.10 | 0.392 | -4.38 (-14.52–5.75) |
|  | Education | 0.15 | 0.164 | 7.26 (-3.01–17.53) |
|  | Comorbidity | -0.05 | 0.648 | -0.51 (-2.72–1.70) |
|  | Living condition | -0.08 | 0.472 | -3.68 (-13.83–6.46) |
|  | Length of stay | 0.10 | 0.385 | 0.21 (-0.26–0.67) |
|  | Days since discharge | -0.09 | 0.398 | -0.04 (-0.12–0.05) |
|  | BMI | -0.01 | 0.943 | -0.03 (-0.98–0.91) |
|  | SPPB | 0.13 | 0.259 | 1.28 (-0.96–3.52) |
|  | Grip strength | 0.11 | 0.313 | 0.29 (-0.28–0.85) |
|  | Berg Balance Scale | -0.01 | 0.944 | -0.03 (-0.78–0.73) |
|  | 6-min walk test | 0.10 | 0.383 | 0.02 (-0.03–0.06) |
| Mental Health | Age | 0.08 | 0.458 | 0.22 (-0.36–0.79) |
|  | Sex | -0.04 | 0.753 | -0.99 (-7.22–5.24) |
|  | Education | 0.14 | 0.216 | 3.95 (-2.36–10.26) |
|  | Comorbidity | 0.04 | 0.735 | 0.23 (-1.13–1.59) |
|  | Living condition | -0.05 | 0.675 | -1.32 (-7.55–4.91) |
|  | Length of stay | -0.04 | 0.719 | -0.05 (-0.34–0.24) |
|  | Days since discharge | 0.05 | 0.624 | 0.01 (-0.04–0.07) |
|  | BMI | 0.02 | 0.839 | 0.06 (-0.52–0.64) |
|  | SPPB | 0.16 | 0.150 | 1.00 (-0.37–2.36) |
|  | Grip strength | 0.04 | 0.701 | 0.07 (-0.28–0.41) |
|  | Berg Balance Scale | 0.07 | 0.557 | 0.14 (-0.32–0.60) |
|  | 6-min walk test | 0.05 | 0.677 | 0.01 (-0.02–0.03) |

SPPB= Short Physical Performance Battery. SF- 36 = the Medical Outcome Study 36 Item Short-Form Health Survey. HRQOL = health-related quality of life. CI = confidence interval. B = unstandardised beta.

Education = postsecondary (yes/no)
